# Abstract of the Proceedings of the Buffalo Medical Association

**Published:** 1876-07

**Authors:** E. N. Brush

**Affiliations:** Secretary


ART. II.—*Abstract of the Proceedings of the Buffalo Medical Association, May* 25, 1876. Dr. E. N. Brush, Secretary.

Dr. Wyckoff, President, in the chair.

Present, Drs. White, Rochester, Folwell, Little, Brecht, Bielby, Fowler, Gould, Miner, Briggs, Howe, Samo, Hopkins, Macneil, W. W. Miner, Gay, Greene, Cronyn, O'Brien, Bartlett, Hauenstien, Mynter and Trowbridge, Dr. Baker, by invitation.

The minutes of the last meeting were read and approved.

The regular essayist, DR. COXE, being absent on account of illness, DR. ROCHESTER moved that he be given further time. Carried.

The Secretary read a communication from the Boston Society of Civil Engineers in reference to a uniform system of weights and measures. DR. HOPKINS moved that it be placed on file. Carried.

DR. FOLWELL read a detailed report of a fatal case of osteo-myelitis of the femur.

DR. ROCHESTER said that the case reported by Dr. Folwell recalled to his mind the discussion held in this Society in reference to the relation of Potts' Disease of the Spine and tuberculosis. At that time the general opinion was that Potts' disease was generally the result of accident, that it was not due to constitutional trouble, and was seldom alone fatal. He reported briefly a fatal case of the disease which recently came under his observation.

Dr. Cronyn said that Dr. Folwell's case was worthy of consideration for another reason than that advanced, that is, that the constitution of the patient was of such a low condition that none of the means adopted were such as to work a cure. The lesson to be learned was that the favorable results were more often due to the good constitution of the patient than to the means employed.

Dr. White reported the particulars of a case where the bones of a fœtus had been discharged from the bladder.

Dr. Rochester moved that Dr. Mynter's paper, (see May No., page 377,) be taken from the table. Carried.

Dr. Rochester referred to the writings of Dr. Bowditch, and said Dr. Bowditch operates for the following reasons:

1. To save life when threatened.
2. To prolong life even when complicated with severe disease.
3. To shorten latent pleurisy.
4. To give temporary relief merely, in absolutely helpless cases.
5. To relieve cases of common pleurisy which do not easily yield to remedies after a few weeks treatment.

Dr. Rochester said that he believed the plan of Dr. Bowditch was one which it was eminently safe to follow. His personal experience had been somewhat extended, having operated on over twenty cases, in one of which he found it necessary to repeat the operation twelve times. In none of these cases had he operated until all other appropriate means had been carefully tested. Dr. Mynter proposes to operate as soon as the diagnosis is made out, the idea being to prevent compression of the lung, dyspnœa, etc. As he understood Dr. Mynter, he would operate at any time as soon as fluid is present; if this shall prove to be good and successful practice he would give his adherence to it, but it seems doubtful. We cannot say of a patient, on the fourth or fifth day of acute pleuritis, that the fluid will not be absorbed in a short time, under appropriate remedies, without dangerous or even troublesome compression of the lung. He did not believe that Dr. Bowditch would advise any one to operate as early as the fifth to the tenth day when but a small amount of fluid was present and no dyspnœa. He did not desire to stand in the way of progress, but care must be taken that what we call progress or improvement is not trifling with human life. To open the pleural cavity to remove a simple serous effusion, when other means had not been faithfully tried, seemed somewhat analagous to converting a simple fracture into a compound one. There were occasions when the fluid accumulated so rapidly as to seriously interfere with breathing, when it would be necessary to employ radical measures to get rid of the effusion, but in a case where there were no symptoms other than those of acute pleuritis, with but little fluid, he did not think there was any warrant for opening into the pleural cavity. Every member of the Association knows that these effusions disappeared frequently under simple treatment, and hence the operation was one which should not be made until other means had been tried.

Dr. Mynter said that the only two patients he had operated on recovered. The first patient was operated on on the sixth day, recovered in sixteen days with no remaining evidence of pleuritis. The second patient was operated on on the twentieth day, three and one-half quarts of fluid being removed. Recovery was perfect on the fourteenth day after. In this case there is present a slight dullness in the lower part of the scapular region. The operation was not one which he had himself advanced, in the hospitals in Denmark it is frequently made, and has been cultivated to a considerable extent. For the last ten years he was connected with a large hospital in Copenhagen as resident physician, and saw at least twenty-five cases operated on with perfect success. He did not see that that there was any danger in the operation with a capillary trocar and a clean apparatus.

Dr. J. F. Miner said that Dr. Mynter's expression in reference to "cultivating an operation" afforded a text for some remarks as to the danger of *operating* always and indiscriminately on all cases. Surgeons should be very careful in exposing their patients to the risks of an operation when recovery was likely to follow other and less radical methods of treatment. The idea of making an operation in a disease which was, in a large majority of cases, amenable to treatment, should be discountenanced. No operation, however slight, is free from danger, and the careful surgeon should weigh well all other means of treatment before resorting to one. Statistics would have to show that a larger proportion recovered under the mode of treatment under discussion than under others before it could receive professional endorsement.

Dr. Gay said that if the means of physical exploration had reached such a point that we can determine to a certainty whether a pint or three pints of fluid was in the pleural cavity, and the patient had difficulty in breathing, and his heart is displaced by the pressure, thoracentisis should be performed. He did not think that any doubt should exist as to the propriety of the operation. If we could return to the practice of twenty-five or thirty years ago and bleed, we should probably have less pleuritic effusion.

After some further discussion of the subject of thoracentisis, which, however, had more reference to empyema and chronic pleuritis, the Association adjourned.

